# Epigenetic regulation of embryonic stem cell marker miR302C in human chondrosarcoma as determinant of antiproliferative activity of proline-rich polypeptide 1

**DOI:** 10.3892/ijo.2015.3054

**Published:** 2015-06-18

**Authors:** KARINA GALOIAN, AMIR QURESHI, GIANLUCA D’IPPOLITO, PAUL C. SCHILLER, MARCO MOLINARI, ANDREA L. JOHNSTONE, SHAUN P. BROTHERS, ANA C. PAZ, H.T. TEMPLE

**Affiliations:** 1 Department of Orthopaedic Surgery, University of Miami Miller School of Medicine, Miami, FL, USA; 2 Interdisciplinary Stem Cell Institute, University of Miami Miller School of Medicine, Miami, FL, USA; 3 Department of Biochemistry and Molecular Biology, University of Miami Miller School of Medicine, Miami, FL, USA; 4 Center for Therapeutic Innovation, Department of Psychiatry and Behavioral Sciences, University of Miami Miller School of Medicine, Miami, FL, USA; 5 GRECC and Research Service, Bruce W. Carter Veterans Affairs Medical Center, Miami, FL, USA; 6 Department of Biomedical Engineering, University of Miami College of Engineering, Miami, FL, USA; 7 Division of Oncology, Department of Medicine, Sylvester Comprehensive Cancer Center, University of Miami, Miami, FL, USA

**Keywords:** proline-rich polypeptide 1, chondrosarcoma, stem cell marker, miR302c, polycomb protein Bmi-1, Nanog, c-Myc

## Abstract

Metastatic chondrosarcoma of mesenchymal origin is the second most common bone malignancy and does not respond either to chemotherapy or radiation; therefore, the search for new therapies is relevant and urgent. We described recently that tumor growth inhibiting cytostatic proline-rich polypeptide 1, (PRP-1) significantly upregulated tumor suppressor miRNAs, downregulated onco-miRNAs in human chondrosarcoma JJ012 cell line, compared to chondrocytes culture. In this study we hypothesized the existence and regulation of a functional marker in cancer stem cells, correlated to peptides antiproliferative activity. Experimental results indicated that among significantly downregulated miRNA after PRP-1treatment was miRNAs 302c^*^. This miRNA is a part of the cluster miR302-367, which is stemness regulator in human embryonic stem cells and in certain tumors, but is not expressed in adult hMSCs and normal tissues. PRP-1 had strong inhibitory effect on viability of chondrosarcoma and multilineage induced multipotent adult cells (embryonic primitive cell type). Unlike chondrosarcoma, in glioblastoma, PRP-1 does not have any inhibitory activity on cell proliferation, because in glioblastoma miR-302-367 cluster plays an opposite role, its expression is sufficient to suppress the stemness inducing properties. The observed correlation between the antiproliferative activity of PRP-1 and its action on downregulation of miR302c explains the peptides opposite effects on the upregulation of proliferation of adult mesenchymal stem cells, and the inhibition of the proliferation of human bone giant-cell tumor stromal cells, reported earlier. PRP-1 substantially downregulated the miR302c targets, the stemness markers Nanog, c-Myc and polycomb protein Bmi-1. miR302c expression is induced by JMJD2-mediated H3K9me2 demethylase activity in its promoter region. JMJD2 was reported to be a positive regulator for Nanog. Our experimental results proved that PRP-1 strongly inhibited H3K9 activity comprised of a pool of JMJD1 and JMJD2. We conclude that inhibition of H3K9 activity by PRP-1 leads to downregulation of miR302c and its targets, defining the PRP-1 antiproliferative role.

## Introduction

Metastatic chondrosarcoma of mesenchymal origin is the second most common bone malignancy and does not respond either to chemotherapy or radiation; therefore, the search for new therapies is relevant and urgent (1–4).

We have described recently that tumor growth inhibiting cytostatic proline-rich polypeptide 1, PRP-1 (galarmin), statistically significantly upregulated tumor suppressor miRNAs, downregulated onco-miRNAs in human chondrosarcoma JJ012 cell line, compared to chondrocyte culture (5). Among significantly downregulated oncomiRNAs in chondrosarcoma JJ012 cell line after the treatment with this peptide was miR302c, downregulated 6.46-fold. miR302-367 cluster has been described to be differentially expressed in mouse embryonic stem cells (mESCs) and human embryonic stem cells (hES cells), as well as in certain tumors, but not in adult mesenchymal stem cells (hMSCs) and normal tissues (6). The gene coding for the cluster miR302-367 is a Pol II gene with a capped and polyadenylated transcription product. The miR302-367 coding gene contains three exons and two introns with alternative splicing, which may or may not include exon 2; the miRNA cluster is located within the first intron. Eight miRNA loci (miR-302b, miR-302b^*^, miR-302c, miR-302c^*^, hsc-3, miR-302a^*^, miR-302d, and miR-367) are located within an ~700-bp region on chromosome 4. Cell biology data indicated that the miR302-367 promoter activity depends on the ontogeny and hierarchical cellular stage. The promoter activity is functional during embryonic development (hESCs, mESCs, and hECCs), but it is turned off later in development (hMSCs and multiple transformed cell lines). Even though the promoter transcriptional activity is restricted to an embryonic stage, the promoter transcriptional activity was reported to be off in not only adult stem cells (hMSCs) but also embryonic cell lines with no stem cell potential (7,8). Emerging evidence suggests that miRNAs also may play an essential role in stem cell self-renewal and differentiation (9). The expression of the miR302/367 cluster rapidly and efficiently reprograms mouse and human somatic cells to an iPSC state without a requirement for exogenous transcription factors (10,11). To maintain self-renewal and pluripotency stem cells have to prevent differentiation and development. It is thought that overexpressed miRNAs from the miR302/367 cluster in stem cells primarily repress development. This study pursued the identification of functional marker in cancer stem cells, mechanism of its epigenetic regulation, correlated to selective antiproliferative activity of PRP-1.

## Materials and methods

### Human chondrosarcoma JJ012 cell line tissue culture

The chondrosarcoma cells JJ012 were cultured in monolayer. The chondrosarcoma cell line JJ012 was provided by Dr Joel A. Block (Department of Rheumatology, Rush-Presbyterian St. Luke’s Medical Center, Chicago, IL, USA), then cultured and propagated in our laboratory. Media consisted of Dulbecco’s modified Eagle’s medium (DMEM/MEM), supplemented with F12, 10% fetal bovine serum (ATCC), 25 μg/ml ascorbic acid, 100 ng/ml insulin, 100 nM hydrocortisone, and 1% penicillin/streptomycin (Sigma-Aldrich). The cells were trypsinized and used either for the rapid cell proliferation assays or for the mRNA extraction and miRNA arrays. The control samples were not treated with the peptide, whereas 10 μg/ml PRP-1 was added to the experimental series. PRP-1 was synthesized in our laboratory.

### Rapid cell proliferation assay

Cell Proliferation kit, EMD Biosciences (QIA127), was used for this assay. Cells were seeded at 5×10^4^ cells/100 μl culture in the multi-well plate and incubated overnight at 37°C in 5% CO_2_ incubator. The actual assay was performed the next day. The rapid cell proliferation assay is based on the activity of mitochondrial enzymes active in viable cells. PRP-1 was added to corresponding wells just after seeding, before overnight incubation. The colorimetric 96-well assay measures the colorful product formazan formed by WST-1 tetrazolium salt cleavage by the mitochondrial dehydrogenases. The formazan formation was then quantified by measuring the change in absorbance at 450 nm in a microplate reader. The activity of mitochondrial dehydrogenases is proportional to the cell number. No washing, harvesting, or solubilization steps are required. In this series of experiments, PRP-1 was added to corresponding wells immediately after seeding and cell attachment followed by overnight incubation according to the manufacturer’s instructions.

### Glioblastoma cellular ATP assay

A-172, T98G, and U87MG glioblastoma cell lines (purchased from ATCC) were cultured in Dulbecco’s modified Eagle’s medium supplemented with 10% fetal bovine serum, 100 U/ml penicillin and 100 μg/ml streptomycin (Life Technologies). Cells were grown for 24 h at a density of 500 cells per well in 384-well plates. IBET-151, PRP peptide, or vehicle controls (DMSO for IBET-151, saline for PRP) were added directly into the wells. Each condition was tested in triplicate. After 72 h, a CellTiter-Glo Luminescent Cell Viability assay (Promega) was performed according to the manufacturer’s recommendations. Briefly, the CellTiter-Glo reagents were added to the wells and the plate was briefly centrifuged. The plate was incubated 15 min at room temperature, and luminescence was read using an EnVision Multilabel plate reader (Perkin-Elmer). Dose-response curves were analyzed in GraphPad prism and fitted using a non-linear regression analysis.

### Gel electrophoresis and western blotting

Upon confluency, the cells were trypsinized and seeded in 6-well cluster dishes at a concentration of 1×10^6^ cells/ml. The experimental samples were treated with PRP-1 in corresponding concentrations, whereas control samples were not treated with the peptide. The cells were incubated for 24 h in a 5% CO_2_ incubator at 37°C. The following day, the cells were washed with ice-cold phosphate-buffered saline. A protease inhibitor was added to the cell lysis buffer (C2978; Sigma-Aldrich, St. Louis, MO, USA) in a 1:100 ratio. The cells were collected with a scraper and centrifuged at 15, 000 × g at 4°C. The supernatant was collected and the protein concentration was measured. The pellets were frozen at −80°C until loading on the gel (20 μg/lane). Polyacrylamide gel electrophoresis and western blotting reagents were supplied by Lonza, Inc. (Allendale, NJ, USA), and all the related procedures followed the company’s protocol. The catalog numbers for the reagents and the suppliers are listed below.

Pager Gold Precast Gels (59502; 10% Tris-Glycine; Lonza, Inc.); ECL reagent (RPN2109; GE Healthcare, Little Chalfont, UK); Western Blocker solution (W0138; Sigma-Aldrich); ProSieve Quad Color Protein marker (4.6-300 kDa, 00193837; Lonza, Inc.); 20× reducing agent for ProSieve ProTrack Dual Color Loading buffer (00193861; Lonza, Inc.); ProTrack Loading buffer (00193861; Lonza, Inc.); ProSieve ProTrack Dual Color Loading buffer EX running buffer (00200307; Lonza, Inc.); ProSieve EX Western Blot Transfer buffer (00200309; Lonza, Inc.); Immobilon^®^-P Polyvinylidene difluoride membranes (P4188; Sigma-Aldrich).

### Culture of marrow-isolated adult multilineage-inducible (MIAMI) cells

MIAMI cells were grown as previously described (REF: PMID: 15173316). Briefly, whole BM cells were plated at 1×10^5^/cm^2^ in T75 flasks (Costar) in the presence of D-MEM low glucose, 3% FBS, 100 U/ml penicillin (Gibco), 1 mg/ml streptomycin (Gibco). The cells were incubated at 37°C in a 100% humidified atmosphere of 3% O_2_, 5% CO_2_, and 92% N_2_. Half of the medium was changed after a week; thereafter, half the medium was replaced twice a week. MIAMI cells were cultured to 40–50% confluence. For expansion, MIAMI cells were replated at a density of 100 cells/cm^2^ in fibronectin-coated vessels in 95% D-MEM-low glucose, 3% lot-selected FBS, and 100 U penicillin/1,000 U streptomycin (expansion medium) at 3% O_2_, with 50% of the medium changed twice a week.

### MIAMI cell growth assay with PRP-1

MIAMI cells were plated in triplicate at 2,000 cells/cm^2^ in 6-well plates in expansion medium. The following day cells were supplemented with 1, 2, and 10 μg/ml PRP-1. At the end of each assay-day, 7, 10, and 14, 21 cells were rinsed with PBS and detached with trypsin-EDTA, and then counted with a Neubauer hemacytometer chamber.

### Antibodies

Primary: rabbit polyclonal anti-CDK/2 (M2), cat no. sc-1632 Santa Cruz Biotechnologies; mouse monoclonal (9E10) anti-c-Myc, cat no. SC-40 Santa Cruz Biotechnologies; rabbit polyclonal anti-p-c-Myc (Thr58/Ser 62), cat no. Sc-8000R, Santa Cruz Biotechnologies; mouse monoclonal anti-SCML2 (SCMAD14a), cat no. ab51506 Abcam; rabbit polyclonal antip-Src (Tyr416), cat no. 2101S, Cell Signaling; rabbit polyclonal anti-Src antibody, cat no. 2108S, Cell Signaling, rabbit monoclonal anti-p27 Kip1 (D69C12) XP^®^ cat no. 3686; Santa Cruz Biotechnology; mouse monoclonal p21 (F-5), cat no. sc-6246, Santa Cruz Biotechnologies; mouse monoclonal anti Nanog, clone 7F7-1, cat no. MABD24, EMD Millipore; rabbit polyclonal anti-Bmi-1 antibody, cat no. ab38295, Abcam. Mouse monoclonal anti-tubulin, cat no. T5168, Sigma. Secondary: anti-mouse IgG (A9044; Sigma-Aldrich); and goat anti-rabbit IgG peroxidase conjugate (A0545; Sigma-Aldrich).

### Statistical analysis

All experiments were performed in triplicate, and P<0.05 was considered statistically significant. Data analysis was perform using one-way analysis of variance (ANOVA) unpaired t-test (GraphPad Prism; GraphPad Software, San Diego, CA, USA).

## Results

### Comparison of antiproliferative activity of PRP-1 in human JJ012 chondrosarcoma cell line, glioblastoma cell lines and marrow-isolated adult multilineage inducible (MIAMI) cells

This study pursued the identification of functional marker in cancer stem cells, correlated to peptides antiproliferative activity by comparing different cell lines, where miR302-367 cluster either induces or inhibits stemness properties, as well as marrow-isolated adult multilineage inducible (MIAMI) cells that express embryonic stem cell markers. We have previously demonstrated antiproliferative effect of PRP-1 reaching 80–90% inhibition in human chondrosarcoma cells (2,4).

### PRP peptide does not inhibit the growth or viability of glioblastoma cells

**Figure 1:**
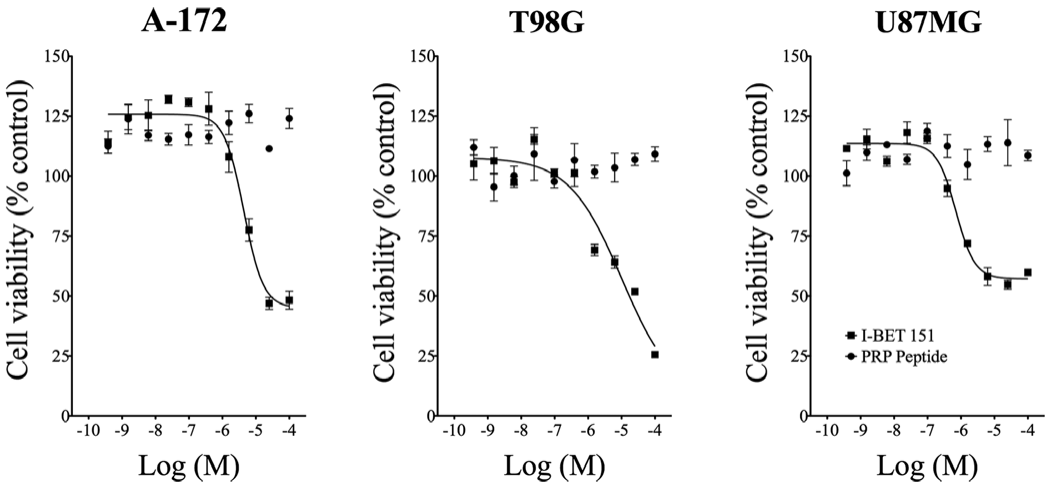
Glioblastoma cell lines A-172, T-98G and U-87. Cells were grown for 24 h at a density of 500 cells per well in 384-well plates. IBET-151, PRP peptide, or vehicle controls (DMSO for IBET-151, saline for PRP) were added directly into the wells. Each condition was tested in triplicate. After 72 h, a CellTiter-Glo Luminescent Cell Viability assay (Promega) was performed.

The BET bromodomain inhibitor IBET-151 reduced glioblastoma cellular ATP levels with potency similar to that we previously reported (IC_50_ = 4.8 μM in A-172 cells, 9.3 μM in T98G cells, and 0.764 μM in U87MG cells) (12,13). In contrast, the PRP peptide showed no effect in any cell line tested, indicating this compound does not reduce glioblastoma cellular proliferation or viability (Fig. 1).

### PRP-1 inhibits MIAMI cells

**Figure 2:**
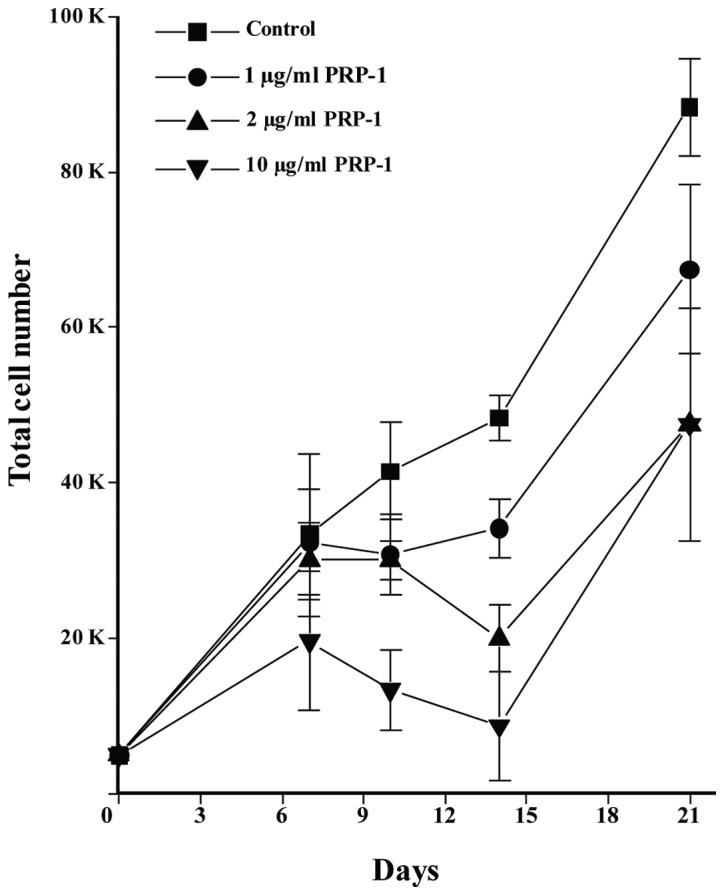
MIAMI cells. Whole bone marrow cells were plated at 1×10^5^/cm^2^ in T75 flasks, MIAMI cells were replated at a density of 100 cells/cm^2^ in fibronectin-coated vessels in 95% D-MEM-low glucose, 5% lot-selected FBS, and 100 U penicillin/1,000 U streptomycin (expansion medium) at 3% O_2_, with 50–60% of the medium changed twice a week.

MIAMI cells resemble primitive stem cells in their capacity to differentiate, at least *in vitro* into mature-like cells from all three germ layers. The expression of embryonic stem cell markers indicate the developmentally immature status of MIAMI cells (14,15). Therefore, it comes as no surprise that the peptide inhibited the growth of these cells. The dose-response inhibitory effect of PRP-1, reaching maximum at 10 μg/ml of the peptide in comparison to untreated control cells is depicted in Fig. 2.

### PRP-1 attenuated the expression of the miR302-367 targets the embryonic stem cell marker Nanog and polycomb protein Bmi-1, while increasing SCML2 expression levels

**Figure 3:**
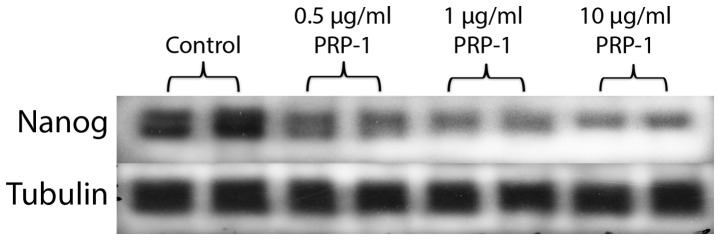
PRP-1 attenuated significantly the expression of Nanog antibody in comparison to untreated control. Mouse monoclonal anti Nanog antibody, clone 7F7-1 was used in 1:1,000 dilution with secondary anti-mouse IgG antibodies. Mouse monoclonal anti-tubulin antibody was used at 1:2,000 and secondary anti-mouse IgG at 1:5,000. Gel exposure time <1 min. Nanog band was detected at 40 kDa and tubulin band at 55 kDa.

**Figure 4:**
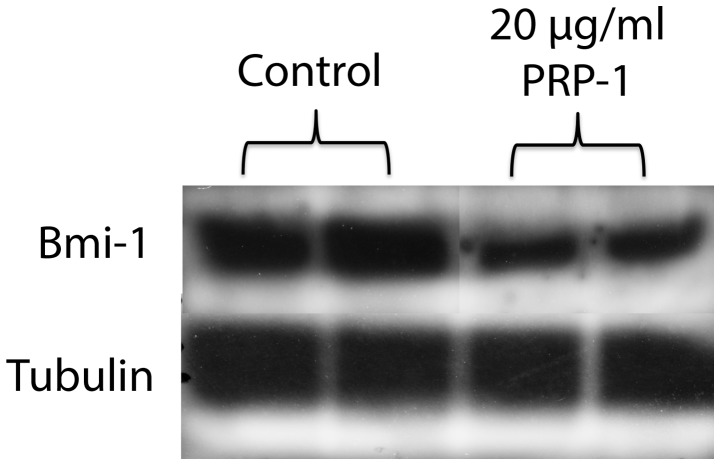
PRP-1 effect of on the expression of Bmi-1 in human JJ012 chondrosarcoma cell line. Rabbit polyclonal anti-BMI antibody was used at 1:1,000 and secondary goat anti-rabbit IgG peroxidase conjugate- at 1:5,000 Bmi-1 bands were detected at 33 kDa. Exposure time, 2–5 min.

**Figure 5:**
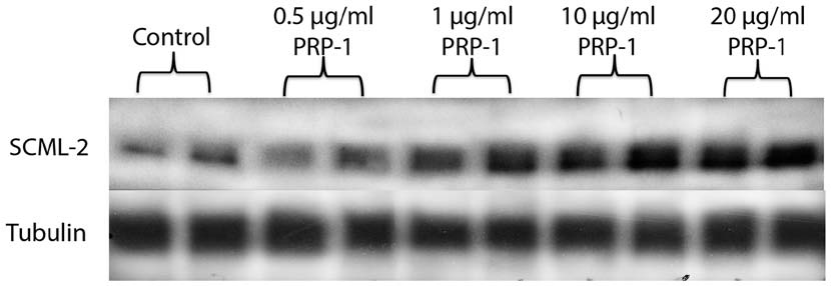
PRP-1 effect on the expression of SCML2 in human JJ012 chondrosarcoma cell line. Mouse monoclonal anti-SCML2 (SCMAD14a), was used in 1:1,000 dilution, and secondary anti-mouse IgG at 1:5,000. Band was detected ~100 kDa region. Film exposure time, 2–5 min. All the antibody dilutions were made with Western Blocker solutions. Mouse monoclonal anti-tubulin antibody was used at 1:2,000 and secondary anti-mouse IgG at 1:5,000. Tubulin band was detected at 55 kDa.

The embryonic stem cell marker Nanog is one of the targets for miR302-367 cluster and it is expressed in many cancers. Nanogs expression was substantially decreased in human JJ012 chondrosarcoma cell line after the treatment with PRP-1 (Fig. 3). The polycomb protein Bmi-1 is also a target for the miR302-367 cluster. Treatment with PRP-1 (20 μg/ml) resulted in strong attenuation of Bmi-1 expression level in comparison to untreated control. Tubulin is demonstrated here as housekeeping protein (Fig. 4). On the contrary, SCML2 expression was increased by PRP-1 in a dose-response manner. SCML2 is not a direct target for miR302-367 cluster, but it is known to repress transcription and is considered as tumor suppressor (Fig. 5).

### PRP-1 decreased c-Myc, p-c-Myc and Src, but not p-Src levels

**Figure 6:**
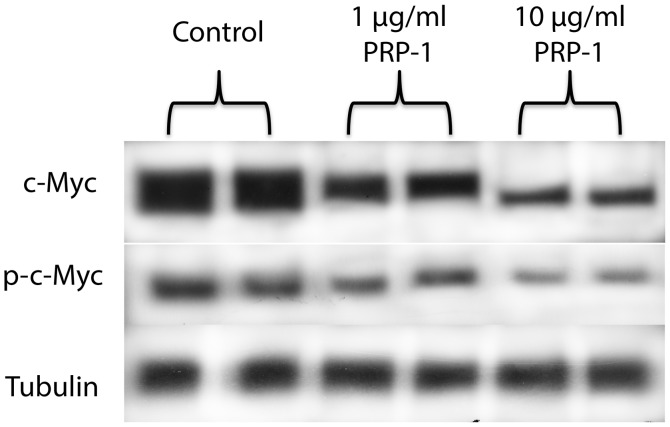
Effect of PRP-1 on c-Myc and p-c-Myc. Mouse monoclonal (9E10) anti-c-Myc and rabbit polyclonal anti-p-c-Myc were used at 1:1,000 dilution, and secondary anti-mouse IgG and goat anti-rabbit IgG peroxidase conjugate at 1:5,000. Band was detected ~67 kDa. Film exposure time, 2–5 min.

Western blot analysis revealed that PRP-1 reduced c-Myc (oncogene target for miR302c) and phosphorylated p-c-Myc expression (Fig. 6).

**Figure 7:**
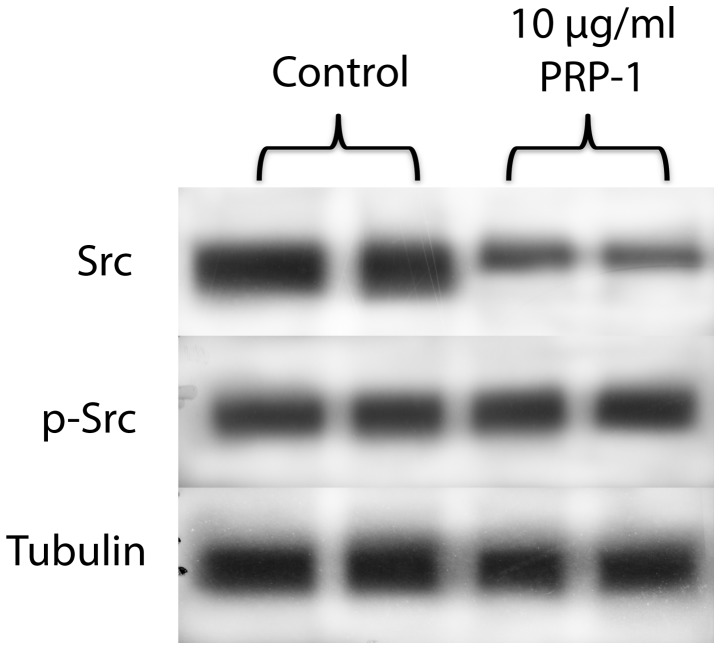
Effect of PRP-1 on Src and p-Src. Rabbit polyclonal anti-p-Src (Tyr416) and rabbit polyclonal anti-Src antibodies were applied to the membranes at 1:1,000, whereas goat anti-rabbit IgG peroxidase conjugate was used at 1:5,000. Mouse monoclonal anti-tubulin antibody was used at 1:2,000 and secondary anti-mouse IgG at 1:5,000. On both gels tubulin band was detected at 55 kDa.

The peptide was tested for its effect on the other oncogene, Src (albeit, its not the target for miR302c) and its phosphorylated form. PRP-1 decreased Src protein levels, but not p-Src expression (Fig. 7).

### PRP-1 effect on cell cycle regulatory proteins p27, p21 and CDK2

**Figure 8:**
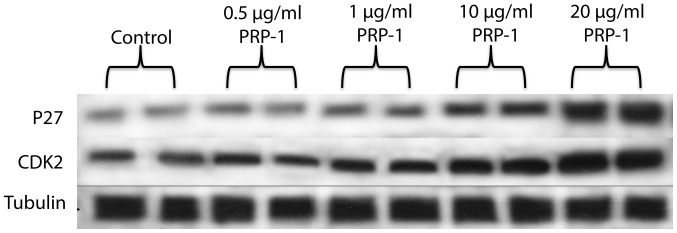
Effect of PRP-1 on the expression of p27 and CDK2 in human JJ012 chondrosarcoma cell line CDK2. Rabbit monoclonal anti-p27 Kip1 (D69C12) and rabbit polyclonal anti-CDK/2 both were used at 1:1,000 dilution in western blocker solution, whereas secondary antibodies goat anti-rabbit IgG peroxidase conjugate was used at 1:5,000. P27 band was detected at 27 kDa and cdk2 at 37 kDa.

**Figure 9:**
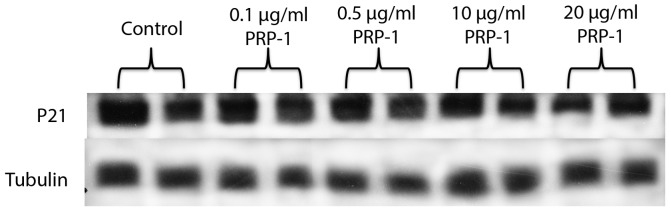
Effect of PRP-1 on the p21 (detected at 21 kDa) expression, in human JJ012 chondrosarcoma cell line. Mouse monoclonal p21 (F-5), was applied at 1:1,000 dilution, and secondary anti-mouse IgG at 1:5,000 dilution. Mouse monoclonal anti-tubulin antibody was used at 1:2,000 and secondary anti-mouse IgG at 1:5,000. Tubulin band was detected at 55 kDa. Film exposure time, 2 min.

It was important to check the expression of cell cycle regulatory proteins with or without the peptide treatment. PPP-1 increased the expression levels of p27, and CDK2 (Fig. 8). P21 expression was reduced after the treatment with PRP-1 in a dose-response manner (Fig. 9).

## Discussion

In the present study, we have demonstrated that miR302c, part of miR302-367 cluster, downstream factor of the embryonic stem cell regulation network determined the antiproliferative activity of cytostatic PRP-1. The cluster is known as a potential stemness regulator in human embryonic stem cells and its expressed in certain tumors but not in adult hMSC and normal cells. Metastatic chondrosarcoma of mesenchymal origin is the second most common bone malignancy and does not respond either to chemotherapy or radiation; therefore, the search for new therapies is relevant and urgent. We described recently that tumor growth inhibiting proline-rich polypeptide 1 (PRP-1, galarmin) significantly upregulated tumor suppressor miRNAs, downregulated onco-miRNAs in human chondrosarcoma JJ012 cell line, compared to chondrocyte culture. Among the miRNAs was miRNA302c^*^ 6.46-fold downregulated after PRP-1 (10 μM) treatment (5). This study pursued the identification of the functional marker in cancer stem cells, correlated to peptide antiproliferative activity and understanding the epigenetic regulation underlying its effect.

We have reported that inhibition of human chondrosarcoma cells, including primary and JJ012 cells with PRP-1 reached >80% (16). In contrast, three cell lines of glioblastoma were not affected by PRP-1 treatment (Fig. 1). Glioblastoma multiforme (GBM) is the most common form of primary brain tumor in adults, often characterized by poor survival. The absence of antiproliferative effect of PRP-1 was expected, taking into consideration that the miR-302-367 cluster is strongly induced during serum-mediated stemness suppression in glioblastoma. Stable miR-302-367 cluster expression is sufficient to suppress the stemness, self-renewal, and cell infiltration within a host brain tissue, through inhibition of the CXCR4 pathway. Furthermore, inhibition of CXCR4 leads to the disruption of the sonic hedgehog (SHH)-GLI-NANOG network, which is involved in self-renewal and expression of the embryonic stem cell-like signature. miR302-367 cluster is able to efficiently trigger a cascade of inhibitory events leading to the disruption of glioma initiating (GiCs) stem-like cells and tumorigenic properties (17). PRP-1 inhibited unique subpopulation of human stromal cells from bone marrow, termed marrow-isolated adult multilineage inducible (MIAMI) cells in a dose- and time-response manner (Fig. 2). These cells are known as the developmentally immature cells with embryonic stem cell markers expression (14,15). They resemble primitive stem cells in their capacity to differentiate at least *in vitro* into mature-like cells from all three germ layers. The observed correlation between the antiproliferative activity of PRP-1 and its effect on downregulation of miR302c, as a stemness marker, explains also the opposite effects of the peptides on the upregulation of proliferation of adult mesenchymal stem cells (MSC), and 2-fold inhibition of the proliferation of human bone giant-cell tumor stromal cells (18).

Somatic cells reprogram to an embryonic stem cell (ESC) comparable induced pluripotent stem (iPS) cell state upon forced expression of exogenously delivered transcription factors, however, expression of exogenous miR-302 cluster (without miR-367) is efficient in attaining a fully reprogrammed iPS state. Methyl-DNA binding domain protein 2, (MBD2), is an epigenetic suppressor, blocking full reprogramming of somatic to iPS cells through direct binding to Nanog promoter elements preventing transcriptional activation. When miR-302 cluster (without miR367) was overexpressed, significant increase in conversion of partial to fully reprogrammed iPS cells by suppressing MBD2 expression, thereby increasing Nanog expression was observed (19). PRP-1 inhibited Nanog expression as miR302c target expression both in MIAMI cells (preliminary not shown results) and in JJ012 chondrosarcoma human cells (Fig. 3). Nanog was not the only target for this miRNA cluster to be decreased by PRP-1. Fig. 4 depicted decreased levels of Bmi-1 protein expression after the treatment with the peptide. Bmi-1 is a component of the Polycomb group (PcG) multiprotein PRC1 complex, a complex required to maintain the transcriptionally repressive state of many genes, including Hox genes, throughout the development. Bmi-1 can act as an oncogene that is particularly potent for the initiation of cancer progression. Bmi-1 is a part of the pathways that are deregulated during tumor development, and thus believed to contribute to neoplastic proliferation and cancer stem cell renewal (20–22). Tumor recurrence following treatment remains a major clinical challenge. Evidence from xenograft models and human trials indicates selective enrichment of cancer-initiating cells (CICs) in tumors that survive therapy. Together with recent reports showing that CIC gene signatures influence patient survival, these studies predict that targeting self-renewal, the key ‘stemness’ property unique to CICs, may represent a new paradigm in cancer therapy. Downregulation of Bmi-1 inhibits the ability of colorectal CICs to self-renew, resulting in the abrogation of their tumorigenic potential. Bmi-1 was demonstrated to play a central part in self-renewal of colorectal cancer initiating cells, as cancer cells were reliant on Bmi-1 to sustain growth and clonal maintenance (23). PRP-1 increased the levels of the polycomb protein SCML2 (Fig. 5). Even though it is not a direct target for miR302-367 cluster, SCML2 interacts with ncRNAs through an RNA-binding region (RBR), contributing to the recruitment of PRC1 to target genes and also directly collaborates in gene repression, particularly repressing transcription. The repressive properties of SCML2 are independent of Bmi-1. SCML2 is thought to be a tumor suppressor (24). Upregulation of tumor suppressor function by PRP-1 is a characteristic feature of this peptide (5,25). Among other affected by PRP-1 miR302c targets was c-Myc. c-Myc is known to be sufficient to activate the fraction increase of CSCs and to activate the ES program (26). Previously, we have reported Myc oncogene inactivating effect of PRP-1 in chondrosarcoma in luciferase assay (2,27), this time we were able to demonstrate with western blot experiments that PRP-1 caused downregulation of p-c-Myc and c-Myc (Fig. 6), which indicated the involvement of peptides in the posttranslational modification and stabilization of c-Myc. The effect of PRP-1 was also tested for p-Src and Src (though Src is not a direct target for miR302-367 cluster) (Fig. 7). An elevated level of c-Src tyrosine kinase activity is suggested to be linked to cancer progression by promoting other signals. The evidence underlying this hypothesis is largely based on the observation that both Src protein levels and, to a greater degree, Src protein kinase activity, are frequently elevated in human neoplastic tissues when compared to adjacent normal tissues (28). Certain cancer cells have similar properties to stem cells (29). Both can avoid cell division stop signals and keep dividing to form new cells. In some cases, a subset of cancer cells within some tumors are cancer stem cells. In stem cells, miRNAs are required to bypass the normal G1/S checkpoint for appropriate stem cell renewal. We demonstrated in our previous study (4) that PRP regulates growth rates of chondrosarcoma cell line, causing cells to accumulate in phase S. In our experiments, PRP-1 increased p27 levels in a dose-response manner (Fig. 8). p27 mediates response to growth inhibitory cytokines, has important antiproliferative role and induces differentiation (30). It is very possible that by inducing p27 levels this peptide causes S phase delay, in the manner of Gatifloxacin action in pancreatic cancer cell lines (31). Gatifloxacin did not induce apoptosis but caused an arrest of cells in S and G2-phase in pancreatic carcinoma cells, inducing p21 and p27. In out experiments, however, p21 levels (Fig. 9) decreased upon the peptide treatment. This finding is in accord with the literature data and can be applied to breast cancer as well, where in rat mammary carcinogenesis increased expression of p21(Cip1), associated with decreased expression of p27(Kip1) was observed (32). Interestingly, the cdk2 levels upon the peptide treatment also increased (Fig. 8). It is possible that PRP-1 causes cytosolic mislocalization of p27 and CDK2, which secures its antiproliferative properties similarly to the anti-migratory/invasive effects resembling those of Nodal in trophoblast cells (33). p27 can still perform its antiproliferative function despite being transported out to the cytosol because of the concomitant nuclear export of CDK2. It is generally accepted that CDK2 promotes G1/S transition by phosphorylating and thereby inactivating Rb, resulting in the activation of E2F transcription factors, as these events take place in the nucleus, cytoplasmic mislocalization of CDK2 would render it inactive in promoting cell cycle progression. Moreover, the epigenetic mechanisms, which are involved lead to antiproliferative activity of PRP-1. miR302c expression was reported to be induced by JMJD2 demethylase (34,35) binding in its promoter region and reduces H3K9me2 methylation. JMJD1C knockdown reduces miR-302 expression (36). JMJD1A and JMJD2C are critical regulators of ES cells, their depletion leads to embryonic stem cell differentiation, which is accompanied by a reduction in the expression of embryonic stem cell-specific genes and an induction of lineage marker genes. Our experimental results proved that PRP-1 strongly inhibited H3K9 activity, comprised of a pool of JMJD1 and JMJD2 in human chondrosarcoma (25). Jmjd2c regulates the expression of downstream effector Nanog through demethylation of H3K9Me3 at the promoter regions of Tcl1, Tcfcp2l1, and Zfp57 and positively regulates the expression of these pluripotency-associated genes. Jmjd2c is required to reverse the H3K9Me3 marks at the Nanog promoter region and consequently prevents transcriptional repressors HP1 and KAP1 from binding (33). The presented experimental data demonstrate that PRP-1 substantially downregulated miR302c targets, stemness markers Nanog, c-Myc, and polycomb protein Bmi-1. We conclude that inhibition of H3K9 activity by PRP-1 leads to downregulation of miR302c and its targets, defining the PRP-1 antiproliferative role. The significance of the presented data underlies the correlation between antiproliferative activity of PRP-1 in human metastatic chondrosarcoma cells and other tumors with the expression of stemness inducing miR302c^*^ that can be a predictive marker for this peptide antitumorigenic activity, targeting cancer stem cells. Our future efforts will be focusing on isolating PRP-1 receptors in cancer stem cells, which is of great therapeutic importance, considering that PRP-1 can be quantified in blood (37).
